# Supervised learning for analysing movement patterns in a virtual reality experiment

**DOI:** 10.1098/rsos.211594

**Published:** 2022-04-20

**Authors:** Frederike Vogel, Nils M. Vahle, Jan Gertheiss, Martin J. Tomasik

**Affiliations:** ^1^ Department of Mathematics and Statistics, School of Economics and Social Sciences, Helmut Schmidt University, Hamburg, Germany; ^2^ Department of Psychology and Psychotherapy, University of Witten/Herdecke, Witten, Nordrhein-Westfalen, Germany

**Keywords:** deep learning, data augmentation, resampling, ageing, embodiment

## Abstract

The projection into a virtual character and the concomitant illusionary body ownership can lead to transformations of one’s entity. Both during and after the exposure, behavioural and attitudinal changes may occur, depending on the characteristics or stereotypes associated with the embodied avatar. In the present study, we investigated the effects on physical activity when young students experience being old. After assignment (at random) to a young or an older avatar, the participants’ body movements were tracked while performing upper body exercises. We propose and discuss the use of supervised learning procedures to assign these movement patterns to the underlying avatar class in order to detect behavioural differences. This approach can be seen as an alternative to classical feature-wise testing. We found that the classification accuracy was remarkably good for support vector machines with linear kernel and deep learning by convolutional neural networks, when inserting time sub-sequences extracted at random and repeatedly from the original data. For hand movements, associated decision boundaries revealed a higher level of local, vertical positions for the young avatar group, indicating increased agility in their performances. This occurrence held for both guided movements as well as achievement-orientated exercises.

## 1. Introduction

Naturally, we have an immanent understanding of our own body’s representation, which is called the sense of body ownership. However, this dimension of self-perception can easily be distorted or extended when we are exposed to conflicting multi-sensory stimulation. Early reports in this regard have been proposed by Botvinick & Cohen [1] with the classical rubber hand illusion experiments. Here, participants perceived a rubber hand as their own when both the fake and the real (removed from sight) hands were stroked simultaneously. The rubber hand phenomenon has since been extended [2,3] and analysed in light of the underlying conditions [4,5]; for instance, the effect was shown to vanish when the shape of the rubber hand was obliterated [6]. Attempts to broaden the sensation of embodiment emerged with the implementation of full-body projections allocated by head-mounted displays. Combining visuotactile, visuomotor and visuoproprioceptive information [7], participants experienced full virtual bodies as their own [8–10]. Yuan & Steed [11] combined both virtual and rubber hand settings by repeating the latter with a display-projected arm and found that participants were also experiencing the virtual extremity as their own.

These findings accumulated ideas on how real-to-virtual-self transformation could evoke changes in behaviour and attitudes of oneself. Addressing these questions under the term *Proteus effect*, Yee & Bailenson [12] gained first insight by linking the self-disclosure of a participant (as the main outcome variable) to the attractiveness of the assigned avatar. Furthermore, Yee *et al.* [13] drew connections between the height of the virtual avatar and the confidence level of the participant when undergoing negotiation tasks. Several other experiments addressing this effect have been performed. For example, Peck *et al.* [14], as well as Banakou *et al.* [15], interrelated the projection of a White person into a Black virtual body with a reduction in implicit racial bias. Banakou *et al.* [16] found that adults confronted with virtual kids’ bodies overestimated object sizes and detected changes in attitude. A comprehensive overview of further areas of application was presented by Ratan *et al.* [17].

The given examples advocate the use of virtual reality (VR) settings to examine behavioural and attitudinal changes when facing one’s ageing self. Becoming old is not exclusively a biological process, but it is also empirically proven to be influenced by both positive and negative stereotypes about age and ageing: while a confident view of the later years of one’s being may enable numerous benefits for the latter, negative stereotypes are usually most prevalent [18] and may cause detrimental effects on the health of the ageing person who has internalized these stereotypes [19]. The range of said effects unfolds from subjective well-being [20] and physiological and cognitive functioning [21] to multimorbidity [22] and mortality [23]. Being of a self-reflexive nature, stereotypes about ageing differ from others (such as those about gender or ethnicity), as we already have them in mind at a young age, but they become self-relevant for ourselves once we get older. The ubiquity of this phenomenon motivates engagement with the older self in artificial settings to activate such stereotypes and examine the consequences. Past studies addressing this conception found indications that people veiled by an old avatar had increased saving tendencies [24] and were less prone to delinquent demeanour [25], which was suggestively linked to an enhanced awareness about one’s future. Yoo *et al.* [26] found a slower walking speed during a virtual shopping tour for participants exposed to older avatars, and Reinhard *et al.* [27] obtained similar results for post-interventional real-life walking speed. However, this effect was only present during the first half of movement. Beaudoin *et al.* [28] transferred these settings onto motor imagery (i.e. participants were asked not to physically execute, but to mentally imagine performing walking exercises). They also detected delays in performance time for old avatars embodied by young participants, and these were presumably attributable to negative beliefs about ageing. Experiencing the old self, however, may also lead to a reduction in such beliefs, as the findings by Yee & Bailenson [29] suggest, when they measured a significant decline in negative stereotyping when people were placed into old avatars. However, it is worth mentioning that the studies by Yee & Bailenson [29] and Beaudoin *et al.* [28] were only partially successful in building profound links to underlying ageing stereotypes; in fact, only one of three dependent measures (a word association task) in the study by Yee & Bailenson [29] and one of four relationships (beliefs on balance and performance time) in the study by Beaudoin *et al.* [28] revealed such connections. Especially, no significant correlations between performance time and beliefs on gait speed, strength and flexibility could be drawn. A study by Vahle & Tomasik [30], intending to conflate such stereotypes and physical capacity, examined the differences in activities between young and old avatars. The activities consisted of slightly physically demanding exercises, including a handgrip strength and weight-holding endurance test. Again, the experiments were only partially successful; they found differences in repeated measures of handgrip strength dependent on avatar age and a correlation with domain-matched age stereotypes. Essentially, those subjects who embodied the old-age avatar and who held strong negative age stereotypes concerning physical performance showed a stronger decline in handgrip strength. However, regarding general handgrip strength and endurance, the results were non-significant. In summary, the results of all studies were mostly based on objective measures gathered after (part of) the experiments (e.g. weight-holding or walking time) and pre-post intervention measured age association tests (e.g. implicit association test (IAT) and word association tests), that is, based on observations occurring outside of the actual activity time.

The present study and the methodological approach were designed to address this gap by inspecting the actual body movement patterns obtained in a similar virtual set-up. For this purpose, young students were randomly assigned either a young or an older avatar (control versus experimental condition) and asked to perform simple, guided upper body movements, such as raising their arms or grasping the hands. During the performances, three points in space (head and two hands) were tracked at a frequency of 10 Hz. Tasks were conceived focusing on the perception of the body and range of motion; there was no achievement orientation. As the above findings suggest, one would hypothesize differences between the groups. These could presumably be expressed by the overall less active and firm movements for the experimental condition, as a major typical age-related stereotype is the reduced mobility of the elderly.

The traditional approach to assess such stereotypes would be to calculate one or more summary statistics for each profile and to compare those ‘features’ between groups/experimental conditions, e.g. by means of classical significance tests. However, a substantial issue with such methods is that, because of how they are designed, features can typically only be examined separately; complex interactions between them are not registered. This aspect is particularly crucial with movement patterns, the subject of our study, because (i) they are multivariate (head and both hands), (ii) each profile is three-dimensional by nature, and (iii) due to their complexity, the information contained can hardly be captured by just a few, hand-picked features (such as overall mean values). Therefore, with these arguments in mind, we decided to take a different approach here, and would like the latter to be seen as experimental itself and basis for further discussion in the scientific community. As mentioned, earlier findings examining ageing stereotypes were, to some degree, ambiguous. Also, to avoid such inconsistencies, we opted for a *reverse* approach to analyse our data: first, said movement patterns were classified using supervised learning with respect to the avatar condition. Then, by evaluating classification accuracy, we can see whether there are indeed substantial differences between groups. In order to make sure that the detected differences are no artefact due to optimization and ‘overfitting’ on the training data, algorithms were trained and evaluated on independent datasets. Finally, we also used the algorithms trained on the original data to separate data obtained from a second, independent and somewhat different study. As, e.g. pointed out by Jelizarow *et al.* [31], validation using fresh independent data that were not used in the development phase can avoid overoptimistic conclusions, because this kind of validation automatically corrects the bias induced by the optimization of the methods’ characteristics. Also, it should be noted that the idea of the reverse approach is not entirely new. It has already been used successfully, especially in medical motion contexts [32,33], which substantiates our proposal to analyse the movement data presented here. From a methodological/machine learning perspective, our analyses further provide some novelty: the algorithms are not only applied on complete movement patterns but also on time segments drawn from the available profiles in a randomized fashion. This had two advantages. First, the dataset/sample size is increased in some sense. Second, achieving the desired outcome on randomly chosen sub-intervals would indicate that differences between the groups were not dependent on a specific, underlying activity; instead, the general behaviour during the experiment varied.

The rest of the article is organized as follows. We first introduce our (primary) experimental study. Then, we describe the candidate machine-learning approaches. As the reverse, supervised learning approach is uncommon in this field, we explicitly offer a comprehensive overview of various methods with mathematical details, making the article also accessible for readers without deep, previous knowledge of machine-learning algorithms. Afterward, we present the algorithms’ application to our data, and compare their ability to separate the experimental conditions. In a reproducibility section, results are checked on data from a second study as described above. Lastly, the features of the underlying movements that seem to be responsible for distinguishability are discussed.

With this paper, we are therefore addressing two gaps: on the contextual side, we extend the existing literature by offering new insights into the motion behaviour of virtually aged humans. From a methodological point of view, we offer a novel approach that can enable access to said insights. This approach can act as a means of analysis when classical methods of feature-wise testing do not use the entire information contained in the data, and, in our belief, can only be beneficial for a broadened and deeper understanding of complex datasets.

## 2. Methods I: design of experiment and data collection

### 2.1. Participants

In total, *N* = 72 students (age 19–30, *M* = 23.31, s.d. = 2.80; 57 female and 15 male participants) were recruited to take part in a VR intervention at a lab section of a medium-sized German university. Recruitment was launched through both online and offline advertising around the campus. Psychology students received credit for participating in the study. The exclusion criteria were severe physiological or psychological impairments, epilepsy, regular intake of psychoactive medication, pregnancy or compulsory use of glasses (because of the limited size of the VR headset).

### 2.2. Materials and measures

Both the physical and virtual environments were exactly the same as those in the work of Vahle & Tomasik [30]. Illustrating pictures, including the virtual characters, can be found there.

#### 2.2.1. Laboratory setting and apparatus

Experiments were conducted in a laboratory section at the University’s campus, covering an area of about 5 × 5 m. The virtual environment was built as an almost exact copy of the surrounding real laboratory. In order to help coalesce with their virtual bodies, a mirror was included in the facility, alongside some basic furnishing (a table and a wall-mounted monitor (Acer ET430K, 43”)) geared to provide the participants space for perceiving and moving their real and virtual bodies. Movements (along the *x*-, *y-* and *z*-axes, as well as corresponding rotations along the pitch-, yaw- and roll-axes) were reflected in that mirror. The resemblance of the physical and virtual surroundings was enabled by a laser scan rendered by a professional VR studio. The transition from reality to simulation was achieved using an HTC VIVE Headset (1080 × 1200 pixels per lens), which was paired with two infrared laser trackers attached to the ceiling. The set-up also included VIVE hand controllers. A total of four avatars (two males and two females) were available for occupation, and each gender was represented by a young (estimated age: 25) and an elderly avatar (estimated age: 75). Said avatars were bought online (https://renderpeople.com/de/3d-people/) in an attempt to fit typical students and older people.

#### 2.2.2. Procedure

After completing a demographics questionnaire obtaining information about gender, age and education, the participants were familiarized with the use of the VR equipment and the attributed headset was mounted. They then found themselves inside the virtual laboratory, standing behind a table with both the monitor and the mirror present in their visual field. The latter enabled them to glance at their virtual appearance before the experimental sequence started. In advance of the VR scenario, one of four available virtual avatars of Caucasian appearance was randomly generated by the test administrator in accordance to the participants gender (male/female) and the applying experimental condition (younger/older), and no comments or announcements were made regarding this choice. A more detailed matching of the avatars’ visual features with regard to clothing, body shape or ethnicity exceeded the resources of the research project. In total, *N* = 35 (29 females and six males) participants were exposed to a young avatar (i.e. age-congruent control condition; hereafter also referred to as target or group 0) and *N* = 37 (28 females and nine males) participants to an elderly avatar (i.e. age-incongruent experimental condition; hereafter referred to as target or group 1) of their own gender. The slight imbalance was due to defective tracking data for a few of the participants. The avatar’s dimensions were adjusted to match the participants’ height as a conformable body size was found to increase individuals’ immersion [34]. The actual scenario started by playing audiotaped instructions requesting the participants to thoroughly reconnoitre their sense of embodiment and perform simple movement tasks in a standing position. The instructions started with a final check to make sure that every participant had the same starting position. Afterward, the actual tasks began. For the first 2 min, these involved familiarization and perception exercises (inspecting the virtual body by looking down and in the mirror, grasping their hands firmly, and touching their own arms and body) to stir the virtual immersion. Afterward, the participants followed further standardized audio sequences consisting of ordinary hand and upper body movements, such as waving at the mirror self, stretching and bending the arms in front of and above oneself, hugging oneself, and wiping one arm with the other. Towards the end of the instruction, the participants were encouraged to move around freely with the arms. All exercises were designed to be performed while standing still, but the participants were not prevented from leaving their initial position. As opposed to hand movements, head motions were barely addressed directly during the sequence; therefore, the head acted as an almost static observer of the tasks accomplished by the hands. The total performance time was 8 min and 17 s (as determined by the standardized audio sequences). The participants then left the virtual intervention and finalized the experiment by completing paper-and-pencil questionnaires on presence and body ownership.

#### 2.2.3. Movement data

**Figure 1.**
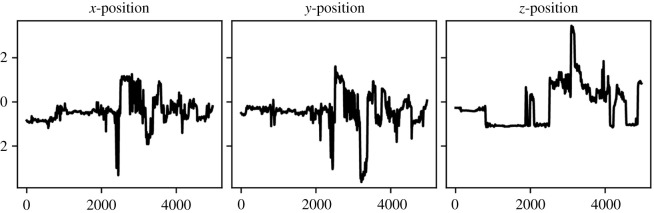
Example of right-hand movement patterns in three axes/dimensions (global positions).

The focus of our analysis was directed toward the movement patterns collected during the procedure described above; these patterns were processed in a reverse fashion by being classified as control versus experimental condition. Movement data were tracked using motion trackers inside the headset and hand controllers, effectively allowing for agreeing movements outside and inside of the VR environment. Both realistic body positions and rotations along three axes (*x*, *y* and *z*) for sojourns in the virtual environment were ensured by precise interpolation. Positional and orientational coordinates were tracked at a frequency of 10 Hz, resulting in patterns consisting of 4970 time points per coordinate. These patterns were then standardized across participants and time points (i.e. the overall mean across all participants and time points was subtracted, and the results were divided by the overall standard deviation acquired across all participants and time points. If the data were split into training and test set (compare *Results section*), standardization was done separately on the training and test data. Positioning was tracked twice: first, as absolute values referring to the global location inside the underlying (laboratory-specific) coordinate system, and second, as local values referring to the position relative to a reference point that depends on the person’s height. This means that there were two coordinate systems: one established by the lab itself (referred to in the following as global or absolute coordinates) and another that was manifested by the participant or, more precisely, by their height (referred to in the following as local or relative coordinates). This is valuable extra information, as there might be cases in which the participants held their hands globally on the same level but individually had to bend or stretch their arms more or less to reach that (global) position. The positions of the head and hands were then calculated in both coordinate systems. Here, the *y*-coordinate referred to the vertical position, the *z*-coordinate to movements towards and away from the mirror (and monitor), and the *x*-coordinate to left-to-right movements (along the mirror/the monitor). Unfortunately, however, some of the tracked data were defective. Therefore, we restrict ourselves to the global and relative positions of the two hands (referred to as features or channels from now on), resulting in a total of 12 channels. Note that the term *channel* is often used when describing the colour content of images in computer vision. Here, we transfer this terminology from two-dimensional images observed over time onto movement profiles that are sequences of coordinates in three-dimensional space. Each of those coordinates can then be seen as a one-dimensional channel. A summary of these channels, as well as a more detailed explanation of the differences between the two coordinate systems, can be found in the electronic supplementary material. Figure 1 shows an example of right-hand (standardized) movement patterns collected during the experiment in terms of the coordinates (global positions) over time in the three-dimensional (*x*, *y*, *z*) space.

### 2.3. Preliminary findings

**Figure 2.**
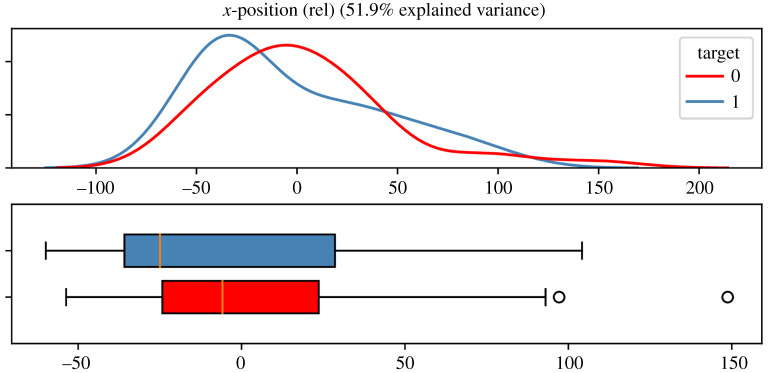
Densities of first PC scores and boxplots for relative *x*-position of the right hand with regard to class affiliation.

As a first step, we used principal component analysis (PCA) as a technique to reduce dimensionality and identify the main directions of variability. The extracted features of the movement profiles were examined separately. An example finding is given in figure 2.

Here, the densities of the first principal component of the relative *x*-positioning of the right hand are displayed as the feature whose explained variance was highest among all (for the first component). However, although it was the maximal amount of variance, it was only approximately 50%. Neither the density plot nor the boxplot revealed first conspicuities. The corresponding Kolmogorov–Smirnov test (*D*(72) = 0.28, *p* = 0.09) and *t*-test (*t*(70) = 0.64, *p* = 0.52) also resulted in non-significant differences between groups. Such preliminary examinations suggested that a simple comparison of single features of whole sessions of the experiments by visual inspection and usual statistical significance tests could not be sufficient to constitute group discrepancies. Therefore, we proceeded with the reverse, machine-learning-based approach, and taking a closer look at subsections. Additionally, this had the advantage of increasing the dataset, as several subsections per movement pattern could be extracted. These segments were of a fixed length but were chosen randomly and inserted into the classifiers described in the next section.

## 3. Methods II: supervised learning

The basic idea of the reverse approach as proposed here is to first train a supervised machine-learning algorithm on a subset of the data (the so-called *training* data). Afterward, the previously trained algorithm can be used to classify the remaining (test) data and the classification accuracy (i.e. the number of correctly classified test units) can be used to judge separability of the groups/experimental conditions. Alternatively, evaluation can be done on test data from a second study. Although there are hundreds of variations of machine-learning algorithms available, for analysing movement patterns with regard to differences as per virtual (age) condition, no standard procedure exists. Therefore, without *a priori* knowledge about the patterns, having a broad field of methods covering a certain range on an *interpretability versus flexibility* scale was necessary. Here, we present five different approaches divided into two categories. Under the term *classical machine learning*, we summarize four methods, namely, linear discriminant analysis (LDA), random forest (RF), support vector machine (SVM) and simple feedforward neural networks (FF), which proceed in a two-step fashion: feature extraction in the first step, and then training on those features in the second step. We also present convolutional neural networks (CNNs) as an automatic *deep learning* technique with implicit feature extraction. As use of those algorithms represents a deviation from traditional, statistical inference (e.g. standard null hypothesis significance tests), we briefly present the mathematics behind the methods and explain the associated (dis-)advantages. For more information, see the electronic supplementary material. Readers with previous knowledge in this area may proceed directly to the *Results* section. All calculations were performed in Python using implementations provided by the Scikit-learn library [35] (LDA, RF and SVM) and Keras [36] (FF and CNN); to visualize our results, we used Matplotlib [37] and Seaborn [38]. Most algorithms need specifications regarding certain hyperparameters. The choices for these will be described in the *Results* section.

### 3.1. Classical machine learning

To classify movement patterns, we first used established approaches from classical machine learning. However, data preprocessing in the form of feature extraction is required to use these tools; for instance, as described in the paragraph above, PCA can serve as an option. The complete procedure is presented in the *Results* section. Regarding notation, the extracted features will be characterized as vectors $x_{i}\in R^{p}$, that is, *x*_*i*_ = (*x*_*i*1_, …, *x*_*ip*_)^*T*^, *i* = 1, …, *n*, where p∈N represents the number of features selected. In (statistical) theory, these samples are interpreted as realizations of a multivariate random variable, which we will denote as *X* = (*X*_1_, …, *X*_*p*_)^*T*^. Each observation *x*_*i*_ occurs with a connected label (a class), which is denoted as $y_{i}\in N$. In the present case, it usually holds that *y*_*i*_ = *k* for *k* ∈ {0, 1} (control versus experimental condition). The number *n* represents the number of training samples (i.e. the data used to fit the classification methods by solving specific optimization tasks as stated below). Predictions for new test samples $x\in R^{p}$ (with true class *y*) are then obtained by following the trained decision rule.

#### 3.1.1. Linear discriminant analysis

LDA is a well-established and popular approach for solving both binary and multi-categorical classification problems given a set of predictors. LDA demands some assumptions from the perspective of statistical modelling, but it provides powerful results when regulations are met. In the case of binary classification, which is our focus here, these requirements expect observations corresponding to class *k* ∈ {0, 1} (control versus experimental condition) to follow a multivariate normal distribution with mean *μ*_*k*_ and covariance matrix Σ (i.e. all classes share the same covariance matrix). Formally speaking, if *y*_*i*_ is the true class of an observation *x*_*i*_, it holds that $N_{p}(\mu_{k},\Sigma )$ is the conditional distribution of *x*_*i*_ given *y*_*i*_ = *k*. With the establishment of these assumptions in Bayes’ formula, the LDA decision rule for assigning a new sample *x* to class 1 can be expressed via

$$x^{T}\Sigma^{-1}(\mu_{1}-\mu_{0})>\frac{1}{2}{\left(\mu_{1}+\mu_{0}\right)}^{T}\Sigma^{-1}(\mu_{1}-\mu_{0})-\log\left(\frac{P(y=1)}{P(y=0)}\right).$$

**Figure 3.**
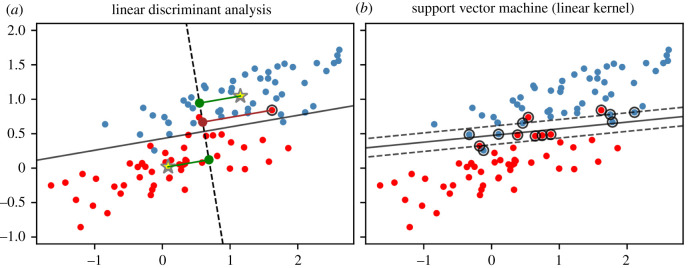
Illustration of the classification concept of LDA and SVM. Data points were simulated having the same covariance matrix and each class is represented equally. (*a*) For LDA, data points and class means (yellow stars) are orthogonally projected (exemplarily displayed as dark red and green lines, respectively) onto the dashed (projection) line. The solid line refers to the corresponding separating line. With prior class probabilities being equal, an observation is assigned to the class whose projected mean is closest to the observation’s projection, e.g. the projection of the circled red point on the right (dark red point) is closer to the projection of the upper class mean. More generally speaking, classifications proceed according to which side of the separating line a data point is located. (*b*) For SVM with linear kernel, the margin (space between dashed lines) around the separating line is maximized while possibly allowing some outliers (circled points; these include all support vectors).

Therefore, for predicting the class of observation *x*, estimates of *μ*_*k*_, Σ and prior class probabilities *P*(*y* = *k*), *k* = 1, 2, are needed. These can be acquired from the (training) data by computing the corresponding arithmetic mean, variance–covariance matrix and the ratio of class-specific and total observations, respectively. Alternatively (but equivalently) to the above decision rule and without distributional assumptions, data can be projected onto a line in $R^{p}$ by building a linear combination *z* = *a*^*T*^*x* [39]. The parameter vector *a* is chosen to maximize the distance between projected class means (the so-called *between-class* variance) relative to the scatter of projected classes (the so-called *within-class* variance) in order to ensure, in some sense, a *minimal* overlap between classes. This optimization procedure creates a cut-point on the projection line where the latter is crossed orthogonally by the line separating the two classes (as far as possible). The exact position of this cut-point thereby depends on prior class probabilities. This projection approach is illustrated in figure 3*a*. Here, a great advantage of LDA is revealed: in the two-dimensional case, the positioning of projection and separating line enables easy interpretation regarding group characteristics. In $R^{p}$, the separating line becomes a hyperplane. LDA is also closely related to logistic regression, another classical and popular approach for binary classification [40].

#### 3.1.2. Support vector machine

SVMs also stem from the idea of classifying data via separating hyperplanes

$$\{x\in R^{p}\;:\;x^{T}\beta +\beta_{0}=0\},$$

where $\beta \in R^{p},\beta_{0}\in R$, which assign classes to feature vectors $x\in R^{p}$ based on the induced decision rule

$$\text{sign}(D(x)),\;\text{with }D(x)=x^{T}\beta +\beta_{0}.$$

Note that now classes are coded by ±1 (e.g. in the situation of the present scenario, class −1 referred to the age-congruent control condition, which we usually declared as class 0). Therefore, *y*_*i*_ ∈ {−1, 1} represents the class corresponding to sample *x*_*i*_. Clearly, for separable data, *D* can be chosen in a way that $y_{i}D(x_{i})>0\;\forall i$. As there might be infinitely many hyperplanes fulfilling such a classification task, the hyperplane of choice here is the one that creates the largest margin *M* between (training) observations of the two classes. However, if classes do not allow perfect separation, one has to adapt such an optimization task by introducing so-called slack variables *ξ*_*i*_, *i* = 1, …, *n*, representing the proportion to which samples are allowed to be off from their actual margin boundary. Under standardization assumptions, it can be calculated that M=1/‖β‖, and the overall optimization task is often expressed as

$$\beta ,\beta_{0}^{\min}\frac{1}{2}{\left\|\beta \right\|}^{2}+C\sum_{i=1}^{n}\xi_{i},\;\text{subject to}:\;\left\{ \begin{array}{c} y_{i}(x_{i}^{T}\beta +\beta_{0})\geq 1-\xi_{i},\\ \xi_{i}\geq 0\;\forall i. \end{array}\right.$$

The parameter *C* is referred to as *cost*, and it controls penalization of outliers. On the one hand, when *C* is large, the optimization procedure will lead to a separating hyperplane that accepts fewer misclassifications and/or data points within the margin than in the case of a small *C*. On the other hand, the margin will be larger for a smaller *C*.

It can be shown that after the minimization task is solved, the decision function is of the form

$$\hat{D}(x)=\sum_{x_{i}\in \Omega }{\hat{\alpha }}_{i}y_{i}\langle x,x_{i}\rangle +{\hat{\beta }}_{0},$$

for some real-valued ${\hat{\beta }}_{0}$, $C\geq {\hat{\alpha }}_{i}>0$, *i* = 1, …, *n*, standard scalar product 〈*x*, *x*_*i*_〉 = *x*^*T*^*x*_*i*_, and the predicted class matches the sign of the function’s result. The parameters of $\hat{D}$ can be acquired by solving the Lagrangian dual of the above optimization task using quadratic programming algorithms. Note that the above sum is only formed by the set of so-called support vectors Ω, the samples lying within the range of the margin and/or the misclassifications. Therefore, a presumably small set of samples fully specifies the decision boundary. In contrast to LDA, SVM is therefore relatively robust to the behaviour of samples located far away from the separating line or hyperplane. For LDA, as described above, the means and covariance matrices of all observations within each class have to be considered. Figure 3*b* depicts the linear SVM decision boundary (plus the margin) with the corresponding support vectors. In the two-dimensional case, as it holds for LDA, the separating line allows for easy interpretation.

However, a linear function as shown in figure 3 may not be appropriate for separating the samples distinctly. In the case of SVM, we can replace the inner product from above with a so-called kernel *K*(*x*, *x*_*i*_), for example, a radial basis as given by

$$K(x,x_{i})=\exp(-\gamma \;{\left\|\;x-x_{i}\;\right\|}^{2}),$$

where *γ* > 0 is a parameter describing the *influence* of a training support vector *x*_*i*_. A small value leads to high variance, and the class of *x*_*i*_ has a strong impact on the decision of assigning a new sample *x* to that class. The kernel function *K* is chosen in advance to consider the shape of the data. With the establishment of different kernels, SVM therefore exhibits some flexibility toward nonlinear data structures.

#### 3.1.3. Random forest

**Figure 4.**
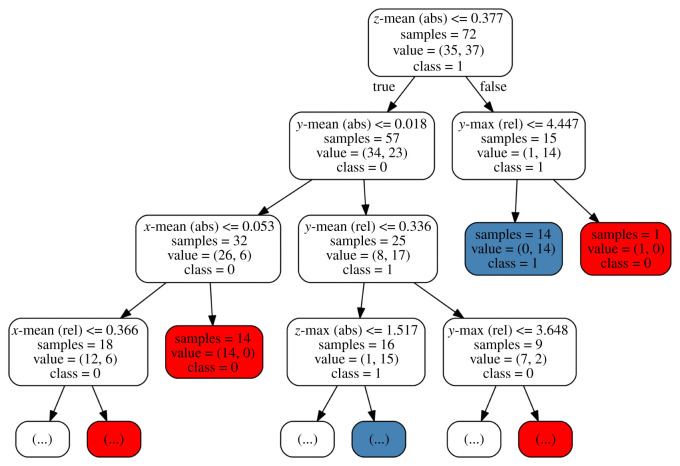
Concept of a decision tree.

Another very popular, yet in contrast to LDA and (partially) SVM, nonlinear approach for classification tasks involves RFs that originate from the simple construct of decision trees. The latter divide the predictor space (i.e. $R^{p}$) into *J* distinct regions *R*_1_, …, *R*_*J*_ and assign each (test) observation to the class that appears most frequently (among training observations) in the region where it belongs. Figure 4 illustrates this concept using some specific features of the present study.

Here, means and maxima were calculated for each channel of the right hand (i.e. absolute and relative *x*, *y* and *z*-positions) of all 72 available movement patterns, disclosing 12 basic features for the tree to make decisions about group affiliation. The first entry in each box denotes the split condition. If it is fulfilled, a sample moves along the left branch of the tree; if not, the right branch is considered. The second entry denotes the number of samples at a certain node, and the third entry (*value*) reveals the class affiliations for that number. The currently dominating class is denoted as the fourth entry. To find the matching class for a (test) observation, the latter moves down the tree as conditions demand; when an endpoint (a leaf/final node) is reached, class affiliation is set by the most frequent class in that node as training observations constituted beforehand. This procedure can be repeated for a number of *m* trees; the overall prediction is then determined by a majority vote. Obviously, a decision tree enables great interpretability, as we can directly extract which and how predictors determine class predictions. In the case of RFs, decision trees are considered in a two-level randomized fashion: First, trees are built on bootstrapped training samples, which means that *new* samples of the same size are generated from the original ones by drawing observations with replacement from the latter. Furthermore, when splits are performed, only a random sample of *r* < *p* predictors is considered split candidates. Due to RFs’ ensemble structure, the profound interpretability of single decision trees is somewhat lost when expanding into such an RF. However, the prediction accuracy is usually much better for a whole forest. Reconciling the loss of interpretability, it is also possible to assess variable importance. A deeper insight into these advantages and limitations of RF methods with a focus on psychological research can be found in the work of Strobl *et al.* [41].

#### 3.1.4. Feedforward neural network

**Figure 5.**
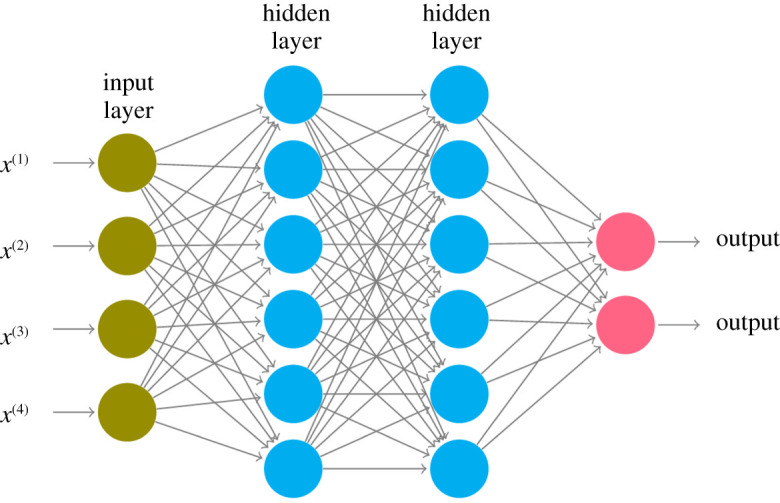
Concept of a simple feedforward network. Elements are the four-dimensional input $x\in R^{4}$ and two hidden layers *F*_1_, *F*_2_ with six neurons each, i.e. functions $F_{1}\;:\;R^{4}\to R^{6}$, $F_{2}\;:\;R^{6}\to R^{2}$, leading into two output neurons.

Feedforward networks consist of name-giving feedforward layers, where each layer is a linear transformation $F\;:\;R^{p}\to R^{q}$ of its input $x\in R^{p}$, that is,

F(x)=Ax+b,

with weight matrix $A\in R^{q\times p}$, bias vector $b\in R^{q}$, and q∈N is the number of so-called neurons in the layer. Both *A* and *b* of the first layer need to be initialized (e.g. by normally distributed values) and are then optimized to fit the task at hand. Therefore, some error function (in our case, categorical cross-entropy) and optimization algorithm (in our case, Adam optimization) to detect minimal loss need to be used. An illustration of a simple network configuration can be found in figure 5.

Here, the network has a four-dimensional input (i.e. four (previously extracted) features), and two hidden layers (blue) with six neurons each. The two output neurons (red) represent probabilities for class affiliations (that is why for a binary classification problem there are two such neurons). Specifically, by using the so-called softmax activation function

$$\psi \;:\;R^{2}\to \{y\in R^{2}|y_{1},y_{2}\geq 0,y_{1}+y_{2}=1\},\;\psi {\left(x\right)}_{j}=\frac{e^{x_{j}}}{e^{x_{1}}+e^{x_{2}}},\;j=1,2,$$

input data are transformed into an output that is in the range [0, 1] and whose entries sum up to 1 (as required for probabilities). Final predictions are then obtained by assigning to the class that corresponds to the neuron entry whose probability is the highest. Feedforward networks provide high prediction accuracy for a broad range of problems [42], including nonlinear situations. However, the increase in flexibility is accompanied by a loss in interpretability: usually, the features that allow certain kinds of predictions are not directly assessable. Here, we mainly included these networks as a less complex consociate of CNNs to explicitly display the differences between the two techniques.

### 3.2. Deep learning with convolutional neural networks

Over the last few years, CNNs have achieved remarkable results for classification tasks, especially regarding computer vision [43] and natural language processing [44]. Originally designed for two-dimensional problems (see Rawat & Wang [45] for an overview of CNN usage for image classification), CNNs are now broadly used for time-series analysis, as well (see Fawaz *et al.* [46] for a general overview or the work by Zheng *et al.* [47] and Cui *et al.* [48] for popular architectures). More specifically, they have been proven to deliver promising results for human activity recognition tasks [49,50]. The motivation behind the deployment of CNNs in these areas emerged from earlier inconvenient methods for time-series classification, which often required laborious hand-crafted feature extraction [51]; thus the need for a technique to automatically learn from time-series data emerged.

**Figure 6.**
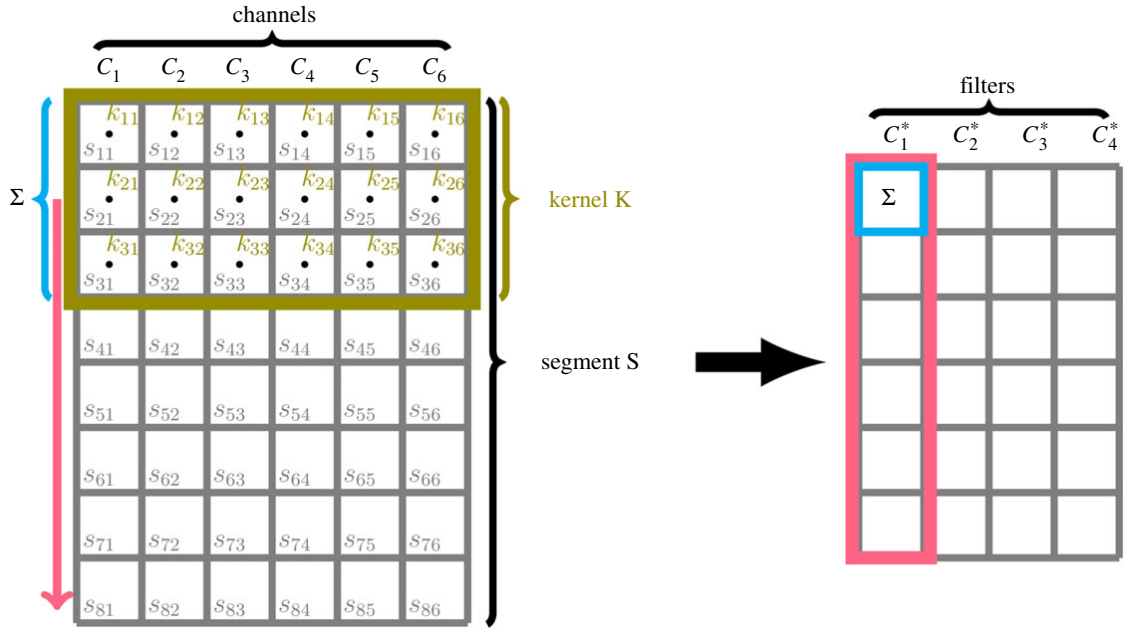
Concept of convolutional layers. A kernel $K\in R^{3\times 6}$ (consisting of entries *k*_11_, …, *k*_36_) with filter length *F* = 3 is slid over the time dimension of a segment $S\in R^{8\times 6}$ (consisting of entries *s*_11_, …, *s*_86_. With the segment’s height of 8, the kernel of size 3 will move along the data over 8 − 3 + 1 = 6 steps. In each step, corresponding components of the kernel and the segment are multiplied and summed up (this operation is denoted by Σ), leading to an output vector of length 6. These operations may be repeated for a number of filters (here: four filters $C_{1}^{*},\ldots ,C_{4}^{*}$).

CNNs usually consist of a combination of so-called convolution and pooling layers. The basic concept of convolution layers is simple: they slide a filter over the time-series data (or part of it) and extract relevant features automatically. If we insert some time segment *S* of size (*W*, *C*), where W∈N denotes the window size (number of time points per segment), and C∈N denotes the number of channels (i.e. the positional global and local *x-*, *y-* and *z*-coordinate, which leads to *C* = 6, when features were considered separately for the left and right hands, or *C* = 12 for both hands), data will be transformed via one-dimensional discrete convolution. The latter is performed for each channel independently, and the outcomes are then summed up. These two steps (convolution and summation over channels) lead to an output of the form

$$(K\;*\;S)(i)=\sum_{c\in \{1,\ldots ,C\}}\sum_{1\leq u\leq F}K(u,c)S(i+u-1,c),\;1\leq i\leq W-F+1,$$

where $K\in R^{F\times C}$ is a convolution kernel (or filter) of length F∈N. Some bias vector may be added to the result. As in the case of feedforward layers, kernel and bias entries have to be initialized, using, for example, the normal distribution. This procedure may be repeated for several kernels/filters (to extract more information) resulting in some output segment (W*, C*), where W* = *W* − *F* + 1 and C* is the number of filters used. A simple illustration of this procedure can be found in figure 6. Afterward, we may transfer the output into a so-called pooling layer which functions as riddance of obsolete information and parameter reduction. For instance, this can be done by considering only the neuron of maximal value in a certain filter range (so-called max-pooling, see electronic supplementary material).

Convolution and pooling can be performed repeatedly in an alternating fashion, which is usually finalized by flattening the output into an element $x\in R^{d}$ (where *d* is the product of the output shape components of the last pooling layer) and further processed via feedforward layers (see above). All occurring weights, again, have to be fitted with regard to the task at hand. Final predictions can be obtained as in the case of feedforward layers, e.g. by closing the network with softmax activation and class assignment according to the largest probability. As no precrafting of features is necessary, CNNs are most flexible and useful as a first assessment of the data at hand. However, they usually do not give insight into their learning process and are therefore not easy to interpret. Given their complexity and the large number of (unknown) parameters involved, CNNs also typically require much richer data than, for example, LDA or (linear) SVM, and a relatively large sample size [52].

## 4. Results

### 4.1. Classification performance

Using supervised learning for classification tasks does not only require the choice of suitable algorithms but also some preparations to make effective use of them, e.g. the choice of training and testing set sizes and optimizers. However, as we do not expect such parameters to highly affect the contextual interpretation of the results, we do not want to go into detail about the related mathematics as we did in the previous section. Readers may refer to the literature, e.g. to Goodfellow *et al.* [53]. The assignment of movement data to the control or experimental group proceeded as follows: we first extracted the mean, standard deviation, minimum and maximum from each channel (summarized under the term *basic features* from now on), resulting in 24 features per body part. Calculations of the features were performed for whole sessions of the experiment, as well as for time segments of different window sizes. For whole sessions, principal components were also used as alternative inputs. To ensure comparability, we also deployed 24 features for such principal component input. Specifically, for each of the six channels/positional coordinates, a separate PCA was performed on the unprocessed participants’ motion profiles; and from each of those six PCAs, the first four principal components were chosen, leading to a total number of 24 features. These features served as inputs for the algorithms listed in the classical machine-learning section; in the case of CNN, the input was given by the pure (standardized) movement patterns. In all cases, the classification procedure was performed by training on 60 and testing on 12 movement sets, each labelled with the corresponding group. Regarding feature extraction, principal components were calculated on training sets, and testing sets were projected into the corresponding space. Although the assignment of a data vector or matrix to training or testing set was conducted randomly, it was ensured that six units of the testing set were labelled referring to target 0 and six units to target 1 (which left 29 and 31 patterns associated with targets 0 and 1, respectively, for the training signals). The classification performance of a pretrained algorithm was then measured by predicting the class affiliations of the test units, and counting the number of correct classifications (i.e. 12 at best). This was performed repeatedly (100 runs, see below), to access a more robust classification accuracy.

#### 4.1.1. Learning and predicting on whole sessions

**Figure 7.**
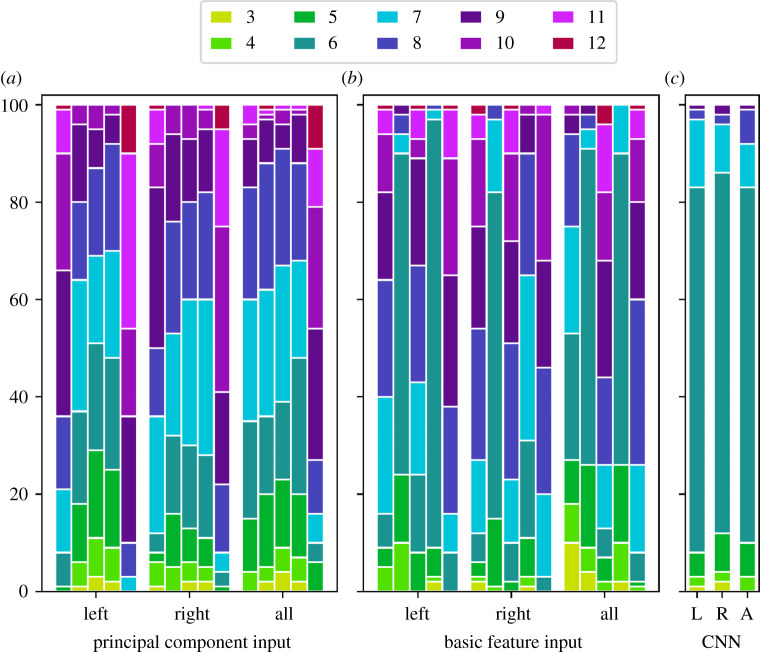
Correct classifications on test data (size 12) in 100 runs based on different inputs. (*a*,*b*) Principal component and basic feature input calculated on whole sessions of the experiment was used. The five bars per body part refer to the classification results by LDA, FF, RF, SVM(RBF) and SVM(LIN), respectively. (*c*) Raw movement data (whole sessions) were inserted into a CNN.

First, we inspected classification rates for whole sessions of the experiment (preprocessed or not as described). With the exception of LDA, all algorithms needed some hyperparameter specification prior to training. For RF, the model was built as an ensemble of 1000 trees, with the current literature recommending to choose a large but computationally feasible number [54]. The number of split candidates was set to $r=\sqrt{\;p}=\sqrt{24}$ (rounded to integer 5), which is the Scikit default setting. For SVM, we compared the usage of a linear (LIN) and a radial basis function (RBF) kernel with parameters *C* = 1 and *γ* = 1/(*p* · Var(*X*)) (again, matching the Scikit default settings). The simple feedforward network used here was a composition of two feedforward layers with 100 neurons each and ReLU activation (see electronic supplementary material for information on activation functions) and a final output layer with softmax activation. The network was trained using an Adam optimizer in 10 training epochs minimizing categorical cross-entropy. Other experimental settings, including a learning rate of 0.01, were set to the default values as provided by the specific libraries. Regarding automatic classification, we established a simple CNN consisting of two convolutional layers with 70 filters (kernel sizes *F*_1_ = 2485 and *F*_2_ = 10, respectively) and a max-pooling layer in between with window size *W*_*M*_ = 3 and stride size *q* = 2. All filters were initialized via a truncated normal distribution. As activation, ReLU was used, and constant bias was added. The subsequent feedforward layer consisted of 100 neurons and used *tanh* activation. Eventually, the final feedforward layer with two neurons and softmax activation was applied. Training proceeded as in the case of FF, with adjusting the learning rate to 0.0001. The total number of trainable parameters can be found in the electronic supplementary material, supplementary material. As input, the (standardized) raw movement data were inserted as whole sequences; no precrafting of certain features was evoked. Referring to the notation (*W*, *C*) of an inserted time segment *S* from the previous section, we have *W* = 4970 (therefore, the size of the first kernel can also be expressed as *F*_1_ = *W*/2). The input shape parameter *C* for the first convolutional layer was predetermined to be *C* = 6 when all features for only one part of the body (right or left hand) were considered or *C* = 12 when the features of both hands were involved. For both networks, the number of layers and also the parameters were not optimized in a data-dependent or other way to avoid distortion of results. However, to build and train a somewhat suitable network architecture with reasonable computational effort, we considered several rules of thumb and popular existing structures [53,55,56]. Figure 7 gives insight into the numbers of correct classifications after conducting 100 repetitions of training and testing (with different participants in the training and testing groups each time) for whole sessions of the experiment.

Strikingly, with PC input, SVM(LIN) and LDA (for single hand movements) achieved far better accuracy rates than the other methods. Specifically, when SVM(LIN) was used, 75% or more (i.e. greater than or equal to 9) of the test observations were classified correctly in approximately 75% or more of all runs. By contrast, FF, RF and SVM(RBF), performed just marginally better than pure guessing. This general superior performance of SVM(LIN) and (partially) LDA transferred to the basic feature case. However, while RF also revealed good results with basic feature input, FF and SVM(RBF) performed as poorly as pure guessing. With deep learning/CNN, results were not more admissible than those of guessing and were therefore not useful in the case of whole session insertion. For both left- and right-hand movements (each separately and combined), approximately 75% of all runs resulted in only six (out of 12) correct predictions with CNN, which means that CNNs are not much better than pure guessing here. These findings, however, are not surprising, as CNNs typically require a relatively large sample size, as mentioned before.

#### 4.1.2. Learning and predicting on segments

**Figure 8.**
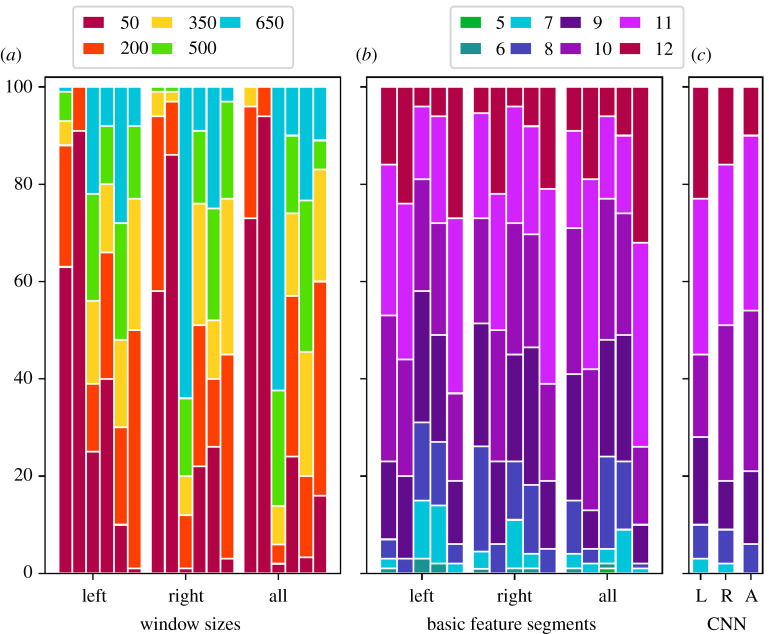
Correct classifications on test data (size 12) in 100 runs based on segmental input. Thirty segments per participant, i.e. 60 × 30 = 1800 for training and 12 × 30 = 360 for testing, were considered. The final prediction for the test set was performed via majority voting per subject. (*a*) Chosen window sizes are displayed. The six bars per body part refer to the sizes chosen by LDA, FF, RF, SVM(RBF), SVM(LIN) and CNN, respectively. (*b*) Basic feature input calculated on segments was used. The five bars per body part refer to the classification results by LDA, FF, RF, SVM(RBF) and SVM(LIN), respectively. (*c*) Raw segmental movement data were inserted into a CNN.

Regarding the classification procedure on segments, a rough grid of different interval lengths, [50, 200, 350, 500, 650], was chosen, and 30 random time sequences of those lengths were drawn from each of the 60 movement patterns selected for the training process (resulting in 1800 training segments). These random segments were allowed to overlap within subjects; also, for each participant, 30 completely new segments were chosen (i.e. for each subject, different parts of the experiment were registered for training). Fivefold (inner) cross-validation was established to determine the optimal window length. Analogously, 30 random segments of the respective size were extracted from each of the remaining 12 test subjects. Classifiers were then used to give predictions about group affiliation on those 12 × 30 = 360 segments. A final prediction for class affiliation for each of the 12 test participants was conducted via majority votes to enable comparability of the performances on whole and sub-sessions. The basic configuration of the classifiers was the same as in the previous subsection. For CNN, the only change occurred for the size (*W*, *C*) of an inserted time segment *S*, which now set *W* ∈ {50, 200, 350, 500, 650}, and the kernel size of the first layer was adjusted to *F*_1_ = *W*/2. Therefore, again, except for the choice of window size, the parameters of this network (see again the electronic supplementary material for the total number of trainable parameters) were not tuned to circumvent distortion of results. The classification rates can be seen in figure 8.

It was highly conspicuous that the segmental classification process elapsed superbly. In general, all methods seemed to perform on an equal level, with SVM(LIN) and FF slightly ahead. When all channels of both hands were inserted, for instance, in approximately 75% of the runs, 11–12 correct predictions were obtained for SVM(LIN). Interestingly, CNN also revealed great results now, coming in third; LDA, RF and SVM(RBF) were slightly in arrears. In summary, however, the number of correct predictions when considering segments was generally on a substantially higher level for all methods than that in the case of whole session evaluation in figure 7. This occurrence can be explained as follows: by choosing 30 segments per participant, the amount of training data was artificially augmented. For the present data, prediction rates increased tremendously for deep learning via CNN and FF (where large sample sizes seem to be particularly important for good performance) and considerably for the other methods. In general, these results did not appear to be affected by the underlying interval length. However, it was striking that when both hand movements were used, LDA and FF seemed to strongly prefer smaller interval lengths, which were scarcely used by SVM(LIN) and CNN. As the only method, RF evoked the highest interval length of 650 time points in more than 50% of the runs for the right and combined hand movements. In the other cases, choices of interval lengths were relatively evenly spread, with no striking patterns to be found. As all algorithms showed good classification results, there was, overall, no indication for limiting the window size to some fixed range. Also, during the training and testing process, we noted that the classification performance itself was relatively robust against window size.

Please note, in our study, segments were allowed to overlap within subjects. Of course, non-overlapping segments could be used as well, which may even lead to (further) improved performance because correlation between segments is reduced. However, the main reason for allowing overlapping segments in our studies was practicability, as those kinds of segments are convenient for handling motion profiles that vary in length between subjects and/or datasets. In particular, in a second study, which we used for checking reproducibility (see the section below), profile lengths were not only different from the study above, but also differing among participants.

### 4.2. Reproducibility

To validate the results presented above, we applied our methods to the study by Vahle & Tomasik [30], which is described below (hereafter referred to as Study 2, with our base study referred to as Study 1).

#### 4.2.1. Outline of Study 2

Data were acquired at the same virtual laboratory section of the same university, ensuring resemblance of environmental parameters and thus enabling comparability solely focusing on the tracked movement data. Furthermore, the technical set-up matched that of the study presented above. However, aside from this environmental resemblance, movements in Study 1 and 2 highly diverged. Study 1 focused on simple, guided movements with no pressure to perform, whereas Study 2 mainly consisted of achievement-orientated and, to some extent, physically demanding exercises. A brief sketch of the second experiment’s essence will be captured first to fully comprehend the differences between the two studies; for details, see Vahle & Tomasik [30]. In total, the movement patterns of *N* = 43 students (age 18–35, *M* = 22.67, s.d. = 3.33, 36 female versus seven male participants) were recorded during a VR intervention. A reduction in the number of participants occurred compared with that in the work of [30] because of missing data. Participants for Study 2 were recruited the same way as for Study 1 through both online and offline advertising around the campus. Again, psychology students received credit for participating. As before, the students faced a randomly assigned virtual avatar of a younger person (age-congruent control condition, *N* = 22 with 18 female and four male participants) or an older person (age-incongruent experimental condition, *N* = 21 with 18 female and three male participants). The beginning of the experiment was constructed similarly as in Study 1 to suffuse the participants with the virtual surroundings. The subjects were again requested to follow a 90 s audio-instructed sequence of familiarization and perception exercises (e.g. as described in the *Methods* section, touching their body). The rest of the experiment differed significantly from the procedure in Study 1, as the subjects were prompted to execute two main physical exercises addressing handgrip strength and endurance. In the first step, regarding handgrip strength, three consecutive measurements with a handgrip dynamometer were performed with 30 s recovery intervals in between while holding the arm in a neutral position with the elbow arched in a 90° angle in front of the body. As suggested by Innes [57], each participant was asked to hold the device with her/his arm in a neutral position, their shoulder adducted and in neutral rotation and their elbow flexed in a 90° angle. To measure the participants’ weight-holding endurance, they were instructed to hold the VR controller for as long as possible in a suspended position directly in front of their body with a straight arm and with a 90° angle between the arm and torso. The test ended when the hand was lowered beneath a point at the lower edge of the sternum. These tasks were only asked to be performed for hands/arms corresponding to the handedness of the participant. Thus, in contrast to Study 1, in which movements were predetermined to a high extent, the subjects’ achievement potential was directly addressed. Physical capacities fluctuated strongly among the participants (the intervention duration lasted from 621 to 1361 s, *M* = 996.54, s.d. = 154.49), and the tracking data size also differed. In contrast to that of Study 1, resolution of recording was set to 1 Hz, leading to a range of 621–1361 for the total number of time points. Again, these included three-dimensional (*x*, *y* and *z*) positions and rotations of three points in space (head and two hands); however, as some of the data in Study 1 were defective, we again only used the global (absolute) and local (relative) positions of each coordinate. These patterns were standardized analogously as in Study 1.

#### 4.2.2. Comparison procedure

**Figure 9.**
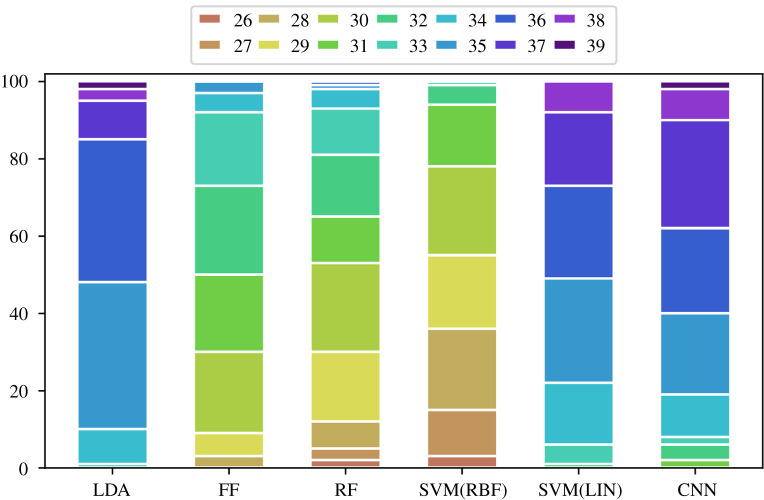
Correct predictions on validation dataset with a total number of 43 participants.

As the focus of Study 2 was the strength and endurance of the hands and arms, a comparison of the hands’ movement patterns in the two studies seems reasonable. In Study 1, motions were predefined, and we simply chose right-hand movements referring to the usually more common handedness (as the actual handedness of the participants was not minuted). To evaluate Study 2, we chose the movement pattern corresponding to the handedness of the participant. In total, only *N* = 4 of the participants were left handed, all of them were female, and only one of them was exposed to an old avatar. To transfer a method pre-trained on Study 1 to Study 2, we proceeded as follows. As the duration of the experiment fluctuated between participants, a classification procedure on segments was preferred to one on whole sessions of experiments, straightforwardly. Furthermore, the results for Study 1 were tremendously better for segmental input, substantiating this approach. Therefore, for training, each of the methods introduced in the *Supervised learning* section was used as described in the *Results* section: 30 random time sequences of a specific interval length were drawn from each of the 72 (right hand) movement patterns available in Study 1. Here, we set the interval length to a fixed size of 200 time points, as our previous results did not indicate a favourable window size. Again, these segments were allowed to overlap, and for each participant, different random segments were selected. The chosen segments were fed into the six classification methods (LDA, FF, RF, SVM(LIN), SVM(RBF) and CNN) configured as before, which were then trained the same way as described in the *Results* section to predict the associated group. For testing, equivalently, 30 random time sequences of length 200 were selected from each of the 43 hand movement patterns collected in Study 2 and inserted into the pretrained classifiers. After the latter conducted their predictions on the chosen segments, majority votes were taken, assigning one class (young or old avatar) to each participant. Figure 9 reveals the number of correct predictions after 100 repetitions of the training and testing procedure.

Note that although the training subjects (72 from Study 1) and test subjects (43 from Study 2) remained fixed here, there was still some variation between runs because of the segments drawn at random in each run. Notably, LDA, SVM(LIN) and CNN achieved remarkably good rates (in most cases approx. 33–37 out of 43, i.e. with an accuracy of approx. 77%–86%) for Study 2, as well. These prediction rates are extremely high, considering Studies 1 and 2 varied substantially in their design: the achievement-related aspect differed tremendously, and the tracking resolution did not match. In summary, results on Study 2 suggest that there are indeed differences in the movement profiles between the two experimental groups. In other words, if there were no substantial differences, but all differences found by the machine learning algorithms in Study 1 were just due to negligible discrepancies or even ‘fitting of noise’, it would have been almost impossible to separate subjects from the second study with such high accuracy.

## 5. Discussion

In this study, we strove to determine differences in behaviour when young people enter virtual characters of different ages. We extended common evaluation routines by inspecting the actual movement patterns collected during the intervention. The analytical approach focused on using supervised learning to assign tracked data to control (young avatar) versus experimental (old avatar) condition. A crucial aspect for this method’s success was the artificial increase in available observations. This was enabled by extracting multiple short time sequences of a complete movement pattern. Linear SVMs especially turned out to be an excellent tool to support the initial assumption that differences in movement between groups indeed exist. A successful transfer to a thematically related but independent study validated this assertion. However, the deeper causes of the observed classification performance have yet to be explored. In the next paragraphs, we hence collect ideas and proposals on how to extract such information.

### 5.1. Interpretability of machine-learning algorithms

**Figure 10.**
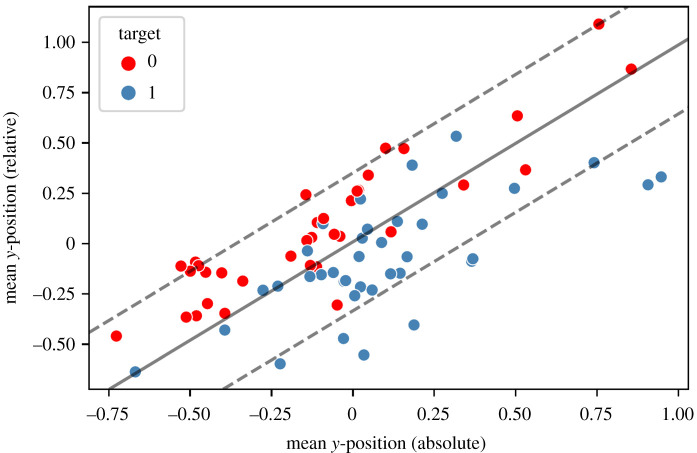
Scatterplots of mean *y*-position (global) versus mean *y*-position (local) for the right hand. All 72 movement patterns (whole sessions) were considered. The solid grey line refers to the decision function of an SVM(LIN) classifier that has been fitted to the two features, the dashed grey lines to the corresponding margin.

The methods used indicated substantial differences in the movements of young adults when they were veiled by either young or old virtual avatars. Deep learning via CNNs, for instance, has the advantage of not requiring the preselection of hand-crafted features, and movement data can be processed automatically. However, which information is learned and processed by the network to arrive at its decisions remains unclear. Luckily, for the present study, this information was, at least to some extent, provided by the very simplistic basic features, namely, the mean, standard deviation, minimum and maximum of the movement patterns. The classification accuracy based on these features is, as demonstrated in the previous chapter, extremely high regardless of the chosen learning algorithm if the latter is applied on a sufficiently large set of randomly chosen time intervals/segments. However, SVM(LIN) (with overall great results) outperformed LDA and FF, which, in turn, surpassed RF and SVM(RBF). As SVM(LIN) and LDA are methods whose decision functions are hyperplanes (the linear discriminant in the case of LDA, and the hyperplanes confining the margin in between them in the case of SVM(LIN)), their classification success insinuates that the feature space may be separable (to some extent) by linear functions/hyperplanes. To pursue this hypothesis, we evoked SVM(LIN) again, entering only two channels at once: the global (absolute) and local (relative) positions of the *x*-, *y-* and *z*-coordinates, respectively. Furthermore, for each of the two chosen channels, only one basic feature at a time (mean, standard deviation, minimum or maximum) was calculated and used for fitting. Here, all 72 movement patterns were used at once, no split in training and testing sets was integrated, and no segments were sampled. The most remarkable conspicuity occurred when considering mean *y*-positions (figure 10). Two things can be observed from inspecting the scatter plot and the SVM decision function: (i) a linear relationship between absolute and relative *y*-coordinate is eminent, which is to be expected because the relative coordinate is mainly just a shift of its absolute correspondent. The size of the shift, however, depends on the participant’s height. Therefore, the dots are still scattered. Nevertheless, (ii) a striking occurrence is revealed. The two classes can be separated very well by a linear function, specifically, the decision function of a linear SVM classifier, in the two-dimensional space of the mean absolute and relative *y*-position. More precisely, for experimental and control participants who have a similar mean absolute *y*-position, the control group sustains a higher level of the relative coordinate. Recapturing the functionality expressed by those coordinate values is necessary to understand these conspicuities: the absolute *y*-position describes the global level of the hands’ location height. Therefore, participants with similar heights hold their hands approximately at the same height. By contrast, the relative *y*-position refers to the level of the hands’ location within the participant itself. Having a higher level of relative *y*-positioning means that the participant has to bend and lift their arms stronger and higher in order to achieve the same level of global height as the participant with lower relative *y*-positioning. For this observation, the height of the participant itself is important to keep in mind. A shorter person typically needs to lift and bend their arms differently from a taller person to reach the same height. Summarizing these considerations, we can conclude that the control group has a higher mean level of bending and lifting their arms, which can be interpreted as an indicator of an overall more active and mobile movement behaviour. The randomized fashion in which time segments were chosen in the previous section substantiates this proposition. Furthermore, as our results in the reproducibility section suggest, this holds true for not only guided movements but also achievement-related situations. The excellent post-transfer classification results indicate that the control group is more engaging during their performances, such as holding their weights, relatively speaking, higher than the experimental group. These indications are in line with the typical age-related stereotype that the elderly are more rigid and less mobile in their movements compared with a young sample, which supports our initial hypothesis. It should be noted though, that this assessment is just one possible/partial explanation for the differences between groups found (see also the section on *Limitations of machine-learning algorithms* below). Further investigating/interpreting those differences will be subject to future research and follow-up studies.

### 5.2. Related work

The technique of boosting prediction performance using segments instead of whole data sequences is an established procedure, as similar techniques (usually called *window slicing*) have been proposed in the literature. Ye & Keogh [58], for instance, presented an algorithm for the univariate case, which extracts some sub-sequences to be maximally representative of a time-series class. Cui *et al.* [48] chose time segments of length 0.9*n* (with *n* denoting the original length of the [longest] time series) mainly to be able to handle time series of different lengths. Zheng *et al.* [47] simply but successively cut sequences into segments of some fixed size. However, these approaches differed from ours, as their choices of segments were not random. Here, to provide evidence that the general behaviour of the two groups differed, selection at random was required (or at least preferable). Another approach of such randomly drawn segments can be found in the work of Möller *et al.* [59]. In their work, functional data were processed by sampling intervals in the functions’ domain and using the corresponding means as predictors in a RF. The main difference to our approach is that in Möller *et al.* [59] those intervals increased the number of predictors/features, whereas in our case, the sample size was increased.

### 5.3. Limitations of machine-learning algorithms

All machine-learning algorithms used provided good or even excellent classification results. However, this was only possible after a data augmentation procedure. It should also be noted that although classes can be separated relatively well by a linear function in the two-dimensional space of the absolute and relative *y*-positions, these two features alone cannot achieve the performance obtained when considering all channels. For example, when the training and testing procedure, as stated in the *Results* section, was repeated for an SVM(LIN) classifier when only inserting segments of the absolute and relative *y*-positions (all four basic features), the results differed from those shown in figure 8. From the latter, it can be seen that with the original SVM(LIN), the best possible outcome of 12 correct predictions was obtained in at least 20% of all runs, and at least 11 correct predictions were obtained in more than 60% of all runs, regardless of the use of the left/right hand only or both. After an input reduction as stated, these numbers dropped; using information on the right hand only, for instance, the numbers dropped to 6% (12 correct predictions) and 34% (11 or 12 correct predictions), respectively. When we used the reduced SVM(LIN) classifier on the validation dataset by Vahle & Tomasik [30], the results were even worse. The best 78% of the original runs revealed 35–38 out of 43 correct predictions (figure 9) and 32 was the minimum amount of accurate decisions; for the reduced version, the best 77% was in the range of 30–33 correct predictions, with 27 being the minimum of such. Apparently, the combination of all (or at least more than two) features is necessary for a reliable classification. Further examinations have to be conducted in order to completely understand and interpret the assignment choices made by the depicted classifiers: for once, other/more sophisticated features with a (potentially) stronger contextual component could be employed (see, e.g. the work by Glowinski *et al.* [60] and Noroozi *et al.* [61] for complex body gesture related features); for another, the methodology could be extended to include more interpretable components [62–64].

### 5.4. Limitations of movement study and implications

In its set-up, the virtual environment allows room for improvement regarding the depth of immersion. Only four pre-built virtual avatars [30] were brought into operation, not resembling the outer appearances (hair colour, clothing, body shape) of the participants, which could have plausibly led to the obstruction of identification. In future work, facial morphing techniques [65] are desirable to integrate facial characteristics. Furthermore, during the experiment runs, some minor technical transmission barriers were encountered. In a few cases, the participants experienced their virtual reflection being delayed or deviating from the movements they initiated (especially concerning elbow and shoulder movements). Additional trackers attached to different body parts might improve the avatars’ actuality and concomitant immersion [34]. They could also allow more insight into which body parts are specifically responsible for the mentioned differences between the young and old avatar groups.

Furthermore, in this study, we only investigated differences in movement patterns. In future work, one might combine the analysis of motion profiles with affective state and (age) stereotype measurements to check for motion differences based on pre-existing stereotypes. As for the participants’ choices, only young students were included, and most of them were female. A repetition of the experiment with elderly participants would be interesting to check for a possible reversion of our findings (i.e. whether older people in young avatars show signs of a higher mobility level).

## 6. Conclusion

Our results give indications that virtual age-flexible character adaptation influences the movement behaviour of young adults. Different supervised learning methods can act as tools for dealing with complex movement data, providing various access points on an interpretability versus flexibility scale. Substantiated by using randomized observation intervals, groups whose avatar represented their age accordingly appeared to give overall more agile and active performances. The established VR framework can easily be altered to obtain further insight into which body parts are specifically involved and to increase the level of immersion.

## Data Availability

All data and source code are available from https://osf.io/rnz62/ [66]. The code used to analyse the data is also available from https://github.com/v0gelf/analyzing_movements [67].
